# Exploration of Relationships among Clinical Gastrointestinal Indicators and Social and Sensory Symptom Severity in Children with Autism Spectrum Disorder

**DOI:** 10.3390/pediatric13040071

**Published:** 2021-11-01

**Authors:** Consuelo M. Kreider, Sharon Mburu, Sanja Dizdarevic, Gerard Garvan, Jennifer H. Elder

**Affiliations:** 1 Department of Occupational Therapy, College of Public Health and Health Professions, University of Florida, Gainesville, FL 32610, USA; sharonmburu@ufl.edu (S.M.); dizsanja@gmail.com (S.D.); ggarvan@ufl.edu (G.G.); 2 Department of Family, Community and Health System Science, College of Nursing, University of Florida, Gainesville, FL 32610, USA; elderjh@ufl.edu

**Keywords:** gut microbiota, sensory processing ASD

## Abstract

Autism Spectrum Disorders (ASD) are associated with co-morbidities such as gastrointestinal (GI) symptomatology, which in the absence of known causes are potential indicators of gut microbiota that may influence behavior. This study’s purpose was to explore relationships among clinical GI indicators—diet, abdominal pain, and stool status—and ASD symptom severity, specifically social and sensory symptoms. Participants were 33 children with ASD, 3 to 16 years. The Social Responsiveness Scale (SRS-2) and the Child Sensory Profile Scale (CSP-2) were used to appraise social and sensory symptomatology. Significant difference was found in overall SRS-2, *t*(31) = −3.220, *p* = 0.003 when compared by abdominal pain status using independent samples *t*-tests. Significant difference was observed for overall CSP-2, *t*(31) = −2.441, *p* = 0.021, when grouped by stool. The three clinical GI variables predicted overall SRS-2 score using multiple linear regression, *F*(3, 32) = 3.257, *p* = 0.036; coefficient for abdominal pain significantly contributed to the outcome. Findings contribute to the growing literature signaling the need to understand occurrence of GI symptomatology more deeply, and in consideration of diet status and its implications in the children’s everyday lives, behaviors, and symptom severity.

## 1. Introduction

Autism spectrum disorder (ASD) refers to a highly prevalent group of heterogeneous neurodevelopmental disorders. ASD is characterized by persistent difficulties in social interactions and communication, as well as restricted and repetitive patterns of behaviors [1]. Restricted behaviors in ASD can manifest as hypo-and hyper-reactivity to sensory input [1]. Altered sensory responses, such as hypo-and/or hyper-reactivity to sensory input, can contribute to behaviors that are difficult for others to understand and predict.

Children with ASD are frequently reported to have picky and limited dietary intake consisting of few fruits and vegetables [2,3,4]. Sensory sensitivities to taste, smell, and food texture and consistency are reported in ASD [5]. Food refusal, as well as self-limited and picky eating behaviors, have been linked to sensory processing differences in children with ASD [5] and are highly correlated with sensory sensitives [6]. It is not uncommon for children with ASD to be on modified diets, whether these diets are therapeutic in nature, such as gluten-free casein-free diets [7] or constrained because of picky eating behaviors [8].

Potential associations have been identified between diet intake and worsened parent-reported gastrointestinal (GI) symptoms and behavioral problems in children with ASD [9,10]. Diet intake has also been shown to influence the composition and regulation of gut microbiota [9,10,11,12], which impacts the brain–gut axis and influences behavior [10,13,14]. Gut microbiota affects mood and behavior via signaling to the brain through multiple physiological pathways [15,16].

Comorbid GI symptomatology of abdominal pain and atypical stool patterns are reported in ASD [9]. GI symptomatology may indicate the status of the gut microbiota in the absence of known etiology [17,18,19]. Children with ASD and comorbid GI symptomatology have been observed to exhibit increased incidence of socially challenging behaviors such as irritability, anxiety, and social withdrawal [20]. However, relationships among social and sensory symptom severity in ASD and diet intake and comorbid GI symptomatology are not yet fully understood.

The purpose of this study was to explore relationships among clinical GI indicators—specifically, parent-reported diet, abdominal pain, and stool status, and the severity of social and sensory behavioral symptoms in children with ASD. Research questions were: (1) Do differences exist in social and/or sensory symptom severity among children with ASD when compared by status of diet, abdominal pain, and stool patterns? (2) Does diet, abdominal pain, and/or stool status predict social and/or sensory symptom severity? We hypothesized there would be differences in the sensory and social symptom severity when compared by the status of each clinical GI indicator, and that social and sensory symptom severity would be greater in the presence of limited diet, abdominal pain, and atypical stool patterns.

## 2. Methods

### 2.1. Research Design

A one-group cross-sectional design was used to address study aims. Oversight was provided by the University of Florida Health Science Center Institutional Review Board (IRB201701140). All parents provided written informed consent and children provided verbal assent when appropriate as based on the child’s age and communication status. Consent and assent were obtained prior to data collection.

### 2.2. Participants

Children with ASD between the ages of 3 to 16 years were recruited for participation through flyers, word of mouth, and community-based primary-care and autism providers, including the University of Florida Center for Autism and Related Disorders. The primary inclusion criterion was a clinical diagnosis of ASD as confirmed by the Autism Diagnostic Observation Schedule (ADOS) and/or Autism Diagnostic Interview, Revised (ADI-R). Potential participants without a confirmatory ADOS or ADI-R were tested by a study investigator who is ADOS and ADI-R research-certified.

### 2.3. Data Collection, Instruments and Variables

All data collection was conducted in person using paper and pencil administration. Parents completed all questionnaires with members of the research team who were present and available to answer questions.

#### 2.3.1. Parent Questionnaire

Parents completed a questionnaire reporting the child’s demographic information and information as to the child’s diet, abdominal pain, and stool patterns. Demographic variables were the child’s age, sex, and race/ethnicity. Predictive clinical GI variables were open-ended questions about diet, abdominal pain, and stool status. The prompt, “Describe your child’s current diet”, was used to gather information about the child’s dietary patterns, preferences, and restrictions. The prompts “Does your child have frequent episodes of abdominal pain?” and “How would you describe your child’s bowel movements or gastrointestinal problems?” were used to query as to the child’s presence of abdominal pain and stool status, respectively. Textual data from open ended responses regarding diet, abdominal pain, and stool status were reviewed with parents and clarified when needed.

#### 2.3.2. Social Responsiveness Scale 2

The Social Responsiveness Scale, 2nd Edition (SRS-2) is a 65-item parent-report measure that identifies the presence and severity of social impairment in individuals with ASD and related conditions aged 2.5 years through adulthood [21]. The SRS-2 has well-established psychometrics. Items are scored from 0 (never true) to 3 (almost always true). The SRS-2 yields an overall *T*-score and subscale *T*-scores. The five subscales include: (1) social awareness, (2) social cognition, (3) social communication, (4) social motivation, and (5) restricted interests and behaviors. Higher item response ratings and higher *T*-scores indicate greater social symptom severity. *T*-scores were calculated using the instrument’s standardized scoring procedures. Subscales were calculated and separately analyzed because of the clinical usefulness of the subscale domains. Missing item responses were accounted for by using the standardized scoring procedure delineated in the instrument manual.

#### 2.3.3. Child Sensory Profile 2

The Child Sensory Profile 2 (CSP-2) is a parent-report measure of sensory processing patterns in children aged 3–14 years [22]. The CSP-2 contains 86 items consisting of statements such as “My child holds hands over ears to protect them from sound.” Each item is rated as follows: 0 = does not apply; 1 = almost never; 2 = occasionally; 3 = half the time; 4 = frequently; and 5 = almost always. Higher scores indicate greater endorsements of sensory-related behaviors.

The CSP-2 has good psychometrics and yields norm-referenced scores for six sensory subscales, three behavioral subscales, and four sensory pattern subscales. Sensory subscales include: (1) auditory, (2) visual, (3) touch, (4) movement, (5) body position, and (6) oral sensory domains. Behavioral subscales include: (1) conduct, (2) attention, and (3) social domains. Sensory pattern subscales include: (1) seeking, (2) avoiding, (3) sensitivity, and (4) registration domains.

Outcome variables from the CSP-2 were ratios from the overall instrument and each subscale. Ratios were calculated by summing the ratings for all items in order to obtain the overall and subscale totals, and then dividing each total by the maximum possible score. Because 17 of 33 participants range (52%) inadvertently omitted responses from item number 86, the item was removed from the calculations of the overall and corresponding (i.e., registration) sub-scale ratios for the entire sample; item 86 was positioned on the questionnaire form in a location that was easily overlooked.

### 2.4. Procedures Regarding Predictive Clinical GI Variables

From the textual data regarding diet, abdominal pain, and stool status, responses were independently categorized by two coders. Prior to coding, the coding team, in conjunction with a senior researcher, established categories for each predictor variable. During the coding process, the coding team and senior researcher discussed potential coding ambiguities to refine and determine eventual classifications.

Diet classifications were: Typical, Therapeutic, and Self-limited. Diet descriptions such as gluten-free, casein-free diets were classified as Therapeutic. Diet descriptions such as “mostly french-fries, chicken nuggets, and bread”, “won’t eat new foods”, and “limited proteins, fruits and vegetables” were categorized as Self-limited.

Abdominal pain was classified as Absent or Present. Stool was classified as Typical or Atypical. Stool descriptions such as “loose”, “hard”, “frequent”, and/or “constipated” were classified as Atypical.

Cohen’s *κ* was used to appraise level of agreement between the two coders. Very good agreement was achieved for Diet (*κ* = 1.00, *p* < 0.001), Abdominal Pain (*κ* = 0.956, *p* < 0.001), and Stool (*κ* = 0.879, *p* < 0.001) categorizations.

### 2.5. Data Analysis

To enable statistical analysis appropriate to the sample size, the three categories for Diet were collapsed into two. The categories of “Therapeutic” and “Self-limited” were combined and used to categorize diets which were limited by the constraints of either the therapeutic diet or the child; this combined category was classified as “Constrained.”

Data were examined for distribution (e.g., sparsity) and tested for outliers and normality. All statistical tests used an *α* = 0.05 to determine statistical significance. Independent samples *t*-tests were used to assess differences, when compared by clinical GI variables, in the normally distributed overall scores for SRS-2 and CSP-2; equal variances were assumed. Because some subscale scores were not normally distributed, Mann–Whitney U tests were used to test association of clinical GI variables on SRS-2 and CSP-2 subscale variables. SRS-2 and CSP-2 overall scores were each modelled separately by the GI variables using multiple linear regression. Fisher’s exact tests were then used to appraise relationships among the categorical variables of Diet, Abdominal Pain, and Stool Status. All statistics were calculated using the software IBM SPSS Statistics for Windows, Version 25, Armonk, New York [23].

## 3. Results

### 3.1. Demographic and Clinical Characteristics of Study Sample

Participants were 25 (75.8%) male and 8 (24.2%) female children with ASD, ages 3–16 years old (mean age = 8.4 years, *SD* = 3.5 years), of which 15 (45%) had typical diet, 24 (73%) had negative patterns of abdominal pain, and 15 had typical stool patterns (45%) (Table 1). The overall SRS-2 score had a median of 79 and an interquartile range of 17. The overall CSP-2 had a median ratio of 0.550 and an interquartile range of 0.196.

### 3.2. Diet and Instrument Scores

No differences were observed in overall scores for both the SRS-2, *t*(31) = 0.247, *p* = 0.806, (typical: *M* = 77.2, *SD* = 14.3; constrained: *M* = 76.3, *SD* = 10.2) and CSP-2, *t*(31) = −0.030, *p* = −0.976, (typical: *M* = 0.54, *SD* = 0.2; constrained: *M* = 0.54, *SD* = 0.1) when compared by diet status of typical versus constrained. Table 2 and Table 3 report comparisons of SRS-2 and CSP-2 subscale scores by diet, abdominal pain, and stool status.

### 3.3. Abdominal Pain and Instrument Scores

A significant difference was found in overall SRS-2 when comparing abdominal pain and no abdominal pain groups, *t*(31) = −3.220, *p* = 0.003, (present: *M* = 86.4, *SD* = 5.6; absent: *M* = 73.1, *SD* = 11.83). No difference was found in overall CSP-2 when compared by abdominal pain grouping, *t*(31) = −1.782, *p* = 0.085, (present: *M* = 0.62, *SD* = 0.12.; absent: *M* = 0.51, *SD* = 0.15).

### 3.4. Stool Status and Instrument Scores

When compared by stool status (i.e., typical versus atypical stool), no difference was found in overall SRS-2, *t*(31) = −1.397, *p* = 0.172 (typical: *M* = 73.6, *SD* = 13.2; atypical: *M* = 79.4, *SD* = 10.7). However, differences were observed for overall CSP-2, *t*(31) = −2.441, *p* = 0.021 (typical: *M* = 0.48, *SD* = 0.1; atypical: *M* = 0.60, *SD* = 0.2).

### 3.5. Predictive Relationships of Diet, Abdominal Pain, Stool Status on Instrument Scores

The three clinical GI variables predicted overall SRS-2 using multiple linear regression, *F*(3, 32) = 3.257, *p* = 0.036. However, only the coefficient for the Abdominal Pain variable significantly contributed to the outcome (Table 4). The multiple linear regression of overall CSP-2 was not statistically significant, *F*(3, 32) = 2.180, *p* = 0.112.

### 3.6. Associations among Clinical GI Variables

As assessed by Fisher’s exact test, there was a significant association between Abdominal Pain and Stool (*p* = 0.021). Of the 18 participants with reported atypical stool patterns, 8 (44%) reported abdominal pain. No significant associations were found among Diet and Stool (*p* = 0.494), and Diet and Abdominal Pain (*p* = 0.697).

## 4. Discussion

This study explored relationships among the clinical GI indicators of parent reported diet, abdominal pain, and stool status, and the severity of social and sensory symptoms in children with ASD. Increased social and sensory symptomatology were found when compared separately by the GI patterns of abdominal pain and atypical stools. Abdominal pain had several linkages to social symptomatology. Areas of increased sensory symptomatology were found in the presence of abdominal pain, as well as when atypical stool patterns were reported. When compared by diet, no differences were found in social or sensory symptom severity (i.e., overall SRS-2, overall CSP-2), with oral sensory symptomatology the most closely approaching significant differences based on diet group. Abdominal pain predicted social symptom severity, with greater symptomatology reported across all SRS-2 domains and overall score. Children with patterns of abdominal pain also reported greater sensory symptomatology in the CSP-2 domains of Avoiding and Touch, which are sensory domains indicative of atypical sensory reactivity [22], specifically hyper-reactivity and under-responsivity, respectively.

As anticipated, the multiple regression of the clinical GI variables (i.e., diet, abdominal pain, and stool status) yielded a significant relationship with overall SRS-2. However, the multiple regression model of SRS-2 scores using the three clinical GI variables was not found to be statistically significant, perhaps due to limited sample size.. Abdominal pain significantly contributed to the SRS-2 outcome, thus providing evidence of its strong relationship to severity of social symptoms. This finding is consistent with other studies that have reported relationships between GI symptoms and severity of specific ASD symptoms, including social withdrawal [20,24], anxiety [20,25], repetitive behaviors and stereotypes [26], expressive language deficits [27], sleep problems [28], and sensory sensitivity [25].

We tested for the existence of relationships among clinical GI variables of diet, abdominal pain, and stool status. The observed relationship between abdominal pain and stool was not surprising as relationships have already been observed between atypical stool patterns, such as constipation and abdominal pain [29]. However, we did not detect a relationship among diet and abdominal pain nor diet and stool status. This finding is inconsistent with other studies that have found dietary intake to influence stool type (e.g., [30]). We suspect our negative finding was impacted by the sample size.

For the children with GI symptomatology of abdominal pain and/or atypical stools, greater social and sensory symptom severity was observed. These findings extend understanding of results from other studies that have examined psychological and behavioral impacts of frequent GI symptomatology—specifically, studies reporting strong correlations among GI symptomatology, sociability, and behavioral symptoms in children with ASD (e.g., [26,31,32]). These findings are also consistent with those from researchers who have found severely restricted behaviors in ASD, such as rigid and compulsive behaviors, to be associated with functional constipation [33] and diarrhea [34]. As prior studies have shown that discomfort associated with GI problems may affect the general state of the child [35], we suspect that discomfort manifested by the presence of GI symptoms, such as abdominal pain or hard stools, may contribute to symptomatology such as reduced social motivation or social communication, as well as increased sensory hyper-responsivity or sensory defensiveness.

For those with atypical stool patterns, greater social symptomatology was reported in the SRS-2 domain of Social Motivation, indicating poorer social motivation than those with typical stool patterns. Those in the atypical stool group also had greater overall sensory symptomatology (i.e., CSP-2 overall score) and in the CSP-2 domains of Registration, Seeking, and Oral. Greater sensory symptomatology in the sensory domains of Registration and Seeking indicate greater sensory hyper-responsiveness; greater symptomatology in the domain of Oral indicates greater amounts of atypical oral sensory reactivity [22]. Our findings support those of Mazurek and colleagues (2013) [25] who found that children with ASD who had various GI problems (e.g., constipation, abdominal pain, bloating, diarrhea, and nausea) lasting three or more months also had higher levels of anxiety and sensory over-responsivity. Sensitivity to the smell, taste, and texture of food can indicate sensory sensitivity or defensiveness [5], which may, in turn, hinder acceptance of foods such as vegetables and fruits—thus contributing to food selectivity and self-limited diets.

The published literature is replete with the functional, social behavioral, and self-regulation impacts of atypical sensory processing in ASD (e.g., [2,36,37]). Sensory processing is widely understood to underpin a broad range of observable behaviors and functional skills [38,39]. Sensory intervention outcome studies (e.g., [40,41,42,43]) contribute evidence of sensory processing’s underlying impact on social, functional, and self-regulation behaviors, including food selectivity behaviors. However, our findings signal a potential relationship among sensory symptomology and diet preferences that may extend existing directional conceptualizations of sensory symptomatology (i.e., sensory impairments’ downstream impact on behaviors [36,38]). While oral-facial sensory impairments can underly restricted food choices, the child’s preferred foods can impact gut microbiota, and in turn GI symptomatology and behaviors, which may include social and sensory behaviors (i.e., sensory symptoms).

Study findings suggest a possible spiraling relationship among ASD symptom severity and a child’s diet that may, via the gut microbiome, have the potential to worsen social and sensory symptom severity. High food selectivity and preference for refined foods are common in children with ASD [44,45]. Sensory impairments in ASD can cause children to choose foods that accommodate sensory sensitivities, thus contributing to observable food selectivity and preference behaviors. These behaviors, may, over time, lead to the overconsumption of specific food types. The foods selected impact the gut microbiota [46], which, in cases of food selections that are low in fiber may induce GI symptomatology such as abdominal pain and discomfort [9,10]. Study findings suggest that GI symptomatology, through gut microbiome pathways, may in turn play a part in worsened ASD symptomatology; albeit interpretation remains limited by the study design and small sample. Future studies should investigate directionality of sensory impairment and ASD symptom severity in consideration of a potential spiraling relationship.

The co-occurrence of GI symptomatology and central nervous system (CNS) symptomatology has been suggested to be linked via alterations in the gut microbiota [47,48], and has been studied across multiple neurological conditions including Parkinson’s Disease, depression, and ASD [49,50]. The possibility of GI symptomatology’s link to CNS symptomatology via the gut microbiota is attributed to complex underlying mechanisms disrupting the neural, immunological, endocrine, and metabolic systems [10,51]. While diet has been identified to play a key role in maintenance of the gut ecosystem [52], it remains unclear the extent to which a limited and restricted diet in children with ASD impacts the gut microbiota. Studies are warranted that examine the degree to which the gut microbiota impacts GI symptomatology and, potentially, ASD symptom severity.

Our study collected food descriptions and feeding behaviors from the parents. We did not, however, examine the nutritional quality, nor the macronutrient composition consumed. Additionally, no considerations were made as to whether the food preference and feeding selectivity were influenced by the families’ food choices and eating preferences [53]. Additional ASD dietary studies are warranted whereby diet preferences—including the family’s preferences, diet—including nutritional content, and the gut microbiota are investigated together. Dietary studies are especially salient because multiple aspects of dietary intake are potentially modifiable behavioral factors that may lead to changes in gut microbiota and may ultimately impact ASD symptom severity. Study findings indicate a range of additional needed studies. However, interpretation remains limited by our small convenience sample that was drawn from one geographical location. The sample was primarily composed of white non-Hispanic (70%) males (76%), which is not representative of racial and ethnic distributions for ASD or for the study location. Additionally, a broad age range was used in this exploratory study. Future studies should include gut microbiota measures, which should consider age, and potentially geographic location, as possible covariables gut microbiota has been shown to vary across developmental age ranges and geographic region [54].

## 5. Conclusions

Overall, we found greater social and sensory symptomatology in the presence of abdominal pain and atypical stool patterns. Study findings signal a predictive relationship among abdominal pain and ASD symptom severity, which may potentially point to gut microbiota status. In conducting this study, we noted that we were the first study to include diet as a variable together with abdominal pain and stool status as potential clinical indicators of gut microbiome status. Study findings contribute to the growing literature signaling the need to more deeply understand occurrence of GI symptomatology in children with ASD (e.g., [55,56,57]), and to understand GI symptomatology in consideration of diet status and its implications in the children’s everyday lives, behaviors, and symptom severity, which in turn impacts parent and family functioning.

## Figures and Tables

**Table 1:** Racial/ethnic, diet, abdominal pain, and stool status distributions of the study sample.

Characteristic	Count (Percentage) *
**Race/Ethnicity**	
White, non-Hispanic	23 (69.70)
Black, non-Hispanic	1 (3.03)
Hispanic	3 (6.06)
American Indian, Eskimo, or Aleutian	1 (3.03)
Asian-Pacific Islander	0 (0)
Other	5 (15.15)
**Diet**	
Typical	15 (45.45)
Therapeutic	7 (21.21)
Atypical	11 (33.33)
**Abdominal pain**	
Absent	24 (72.73)
Present	9 (27.27)
Stool	
Typical	15 (45.45)
Atypical	18 (54.54)

* *N* = 33.

**Table 2:** Comparison of sub-group social symptomatology using the Social Responsiveness Scale 2 (SRS-2) as grouped by Diet, Abdominal Pain and Stool status.

SRS-2 * Variable/Subscale	Diet				Abdominal Pain				Stool			
		Mean Rank Value				Mean Rank Value				Mean Rank Value		
	*p* Value ^†^	Typical *N* = 15	Constrained *N* = 18	95% CI	*p* Value ^†^	Absent *N* = 24	Present *N* = 9	95% CI	*p* Value ^†^	Typical *N* = 15	Atypical *N* = 18	95% CI
SRS-2 Overall	0.806 ^&^	77.23 **	76.28 **	(−7.65, 9.76) ^¤^	**0.003 ^&^**	73.13 **	86.44 **	(−21.7, −4.8) ^¤^	0.172 ^&^	73.60 **	79.39 **	(−14.2, 2.6) ^¤^
Social Motivation	0.442	15.53	18.22	(−14.0, 5.0)	**0.036**	14.85	22.72	(−23, −1.0)	**0.012**	12.43	20.81	(−23.0, 3.0)
Social Awareness	0.401	18.60	15.67	(−6.0, 14.0)	**0.044**	14.94	22.50	(−20, 0.0)	0.421	15.50	18.25	(−14.0, 6.0)
Restricted Interests and Repetitive Behaviors	0.486	18.3	15.92	(−5.0, 8.0)	**0.018**	14.58	23.44	(−17, 0.0)	0.155	14.33	19.22	(−15.0, 0.0)
Social Cognition	0.532	18.17	16.03	(6.0, 12.0)	**0.029**	14.77	22.94	(−21, −1.0)	0.682	16.23	17.64	(−12.0, 7.0)
Social Communication	0.735	16.33	17.56	(−9.0, 7.90)	**0.007**	14.31	24.17	(−21, −3.0)	0.381	15.37	18.36	(−13.0, 4.0)

* SRS-2 = Social Responsiveness Scale 2, ^†^ *p* < 0.05 two tailed, CI = Confidence Interval, ^&^ = *p* values as reported for independent samples *t*-tests, ** = Mean values as reported for independent samples *t*-tests, ¤ = Confidence intervals as reported for independent samples *t*-tests.

**Table 3:** Comparison of sub-group differences in sensory symptomatology using the Child Sensory Profile 2 (CSP-2) as grouped by Diet, Abdominal Pain and Stool status.

CSP-2 * Variable/ Subscale	Diet				Abdominal Pain				Stool			
		Mean Rank Value				Mean Rank Value				Mean Rank Value		
	*p* Value ^†^	Typical *N* = 15	Constrained *N* = 18	95% CI	*p* Value ^†^	Absent *N* = 24	Present *N* = 9	95% CI	*p* Value ^†^	Typical *N* = 15	Atypical *N* = 18	95% CI
CSP-2 Overall	0.976 ^&^	0.54 **	0.55 **	(−0.11, 0.10)^¤^	0.085 ^&^	0.52 **	0.62 **	(−0.21, 0.01) ^¤^	0.021^&^	0.48 **	0.59 **	(−0.21, −0.19) ^¤^
Avoiding	0.630	17.90	16.25	(−0.11, 0.14)	**0.036**	14.85	22.72	(−0.27, −0.01)	0.166	14.40	19.17	(−0.21, 0.04)
Sensitivity	0.532	15.83	17.97	(−0.16, 0.06)	0.094	15.25	21.67	(−0.23, 0.03)	0.062	13.53	19.89	(−0.24, 0.0)
Registration	0.532	18.20	16.00	(−0.08, 0.11)	0.131	15.44	21.17	(−0.20, 0.03)	**0.044**	13.27	20.11	(−0.20, −0.004)
Seeking	0.957	16.87	17.11	(−0.14, 0.14)	0.207	15.67	20.56	(−0.21, 0.06)	**0.016**	12.63	20.64	(−0.27, −0.03)
Auditory	0.817	16.57	17.36	(−0.15, 0.10)	0.166	15.56	20.83	(−0.27, 0.05)	0.464	15.63	18.14	(−0.20, 0.10)
Visual	0.421	18.50	15.75	(−0.07, 0.20)	0.254	15.79	20.22	(−0.27, 0.03)	0.580	15.93	17.89	(−0.20, 0.10)
Touch	0.325	18.87	15.44	(−0.09, 0.20)	**0.036**	14.83	22.78	(−0.31, −0.2)	0.005	11.87	21.28	(−0.30, −0.05)
Movement	0.630	17.93	16.22	(−0.1,0.2)	0.238	15.75	20.33	(−0.25, 0.05)	0.057	13.50	19.92	(−0.27, 0)
Body Positioning	0.682	17.77	16.36	(−0.15,0.2)	0.166	15.56	20.83	(−0.30, 0.05)	0.343	15.20	18.50	(−0.22, 0.05)
Oral	0.073	13.67	19.78	(−0.44,0.02)	0.222	15.71	20.44	(−0.44, −0.09)	**0.044**	13.30	20.08	(−0.44, 0)
Conduct	0.381	18.63	15.64	(−0.07,0.20)	0.254	15.79	20.22	(−0.20, 0.07)	0.030	13.03	20.31	(−0.29, −0.01)
Social Emotional	0.845	17.40	16.67	(−0.14,0.14)	0.094	15.27	21.61	(−0.27, 0.03)	0.166	14.43	19.14	(−0.24, 0.06)
Attention	0.901	17.23	16.81	(−0.14,0.14)	0.890	16.85	17.39	(−0.16, 0.16)	0.135	14.20	19.33	(−0.20, 0.02)

* CSP-2 = Child Sensory Profile 2, ^†^ *p* < 0.05 two tailed, CI = Confidence Interval; ^&^ = *p* values as reported for independent samples *t*-tests, ** = Mean values as reported for independent samples *t*-tests, ¤ = Confidence intervals as reported for independent samples *t*-tests.

**Table 4:** Multiple regression analysis of Diet, Abdominal Pain, Stool Status on Overall Scores for Social Responsiveness Scale 2 (SRS-2) and Child Sensory Profile 2 (CSP-2).

Dependent Variable	Model	*B*	*SE B*	*β*	*t*	*p* *
SRS-2 Overall	Diet	0.259	3.947	0.022	0.066	0.948
	Abdominal Pain	12.950	4.817	0.487	2.688	0.012
	Stool status	0.859	4.321	0.036	0.199	0.844
CSP-2 Overall	Diet	0.097	0.055	0.338	1.781	0.085
	Abdominal Pain	0.051	0.061	0.159	0.842	0.407
	Stool status	−0.007	0.050	−0.024	0.137	0.892

* *p* < 0.05 two tailed.

## Data Availability

The data presented in this study are available upon reasonable request from the corresponding author.
